# Patient perceived changes in sexual dysfunction after initiation of natalizumab for multiple sclerosis

**DOI:** 10.1177/2055217318781989

**Published:** 2018-06-12

**Authors:** Derrick Robertson, Angela Aungst, Ryan Collier, Jhulianna Vivar, Natalie Moreo, Lise Casady, Tuan Vu

**Affiliations:** Department of Neurology, College of Medicine, University of South Florida, USA

**Keywords:** Sexual dysfunction, natalizumab, quality of life, patient-reported outcomes

## Abstract

**Purpose:**

Sexual dysfunction is a common but often overlooked secondary symptom of multiple sclerosis (MS) and can be associated with a decreased health-related quality of life (HRQoL). Natalizumab is a disease-modifying therapy approved for the treatment of relapsing forms of MS. In addition to its efficacy, those using natalizumab have shown improvement in HRQoL parameters, including fatigue and cognition. The idea that improvement in fatigue may also correlate with improvement in sexual dysfunction is the impetus for this study.

**Methods:**

A single-center, open-label, single-arm, 24-week study was performed to evaluate perceived change in sexual dysfunction in MS patients treated with natalizumab. Adults with relapsing MS initiating natalizumab treatment and had a baseline level of sexual dysfunction were enrolled. The primary endpoint was change in the MS Intimacy and Sexuality Questionnaire-19 (MSISQ-19) score from baseline to week 24. Mean age of patients was 41 years, median disease duration was 7 years, and 73% of patients used at least one prior MS disease-modifying therapy.

**Results:**

Natalizumab-treated patients experienced improvement in sexual dysfunction within the first 24 weeks of starting therapy, as demonstrated by the primary subscale of the MSISQ-19 questionnaire (–0.6976, *p* = 0.02).

**Conclusions:**

Given the high prevalence of sexual dysfunction in MS patients and the significant impact it has on HRQoL, more research on this often overlooked symptom of MS could be very informative for patients that are deciding to initiate a new disease modifying therapy.

## Introduction

Multiple sclerosis (MS) is a chronic, immune-mediated disease of the central nervous system (CNS) that affects approximately 2–2.5 million people worldwide.^1^ Approximately 85% of patients present with a relapsing-remitting course, with a majority later developing a secondary progressive course characterized by slow accumulation of disability. This leads to a heterogenic patient population with symptoms involving disturbances of many different systems of the human body including visual, motor, sensory, coordination, balance, bowel, bladder, sexual, and cognitive.

Sexual dysfunction is a common symptom experienced by patients with MS; the estimated prevalence ranges between 40 and 80%.^2^ Sexual dysfunction is often divided into primary (damage directly from MS lesions in areas involving sexual response leading to difficulties with arousal and reaching orgasm), secondary (signs associated with sexual response including motor weakness, muscle spasticity, fatigue and pain related to inadequate lubrication) and tertiary (psychological and sociological impairment).^3^ Sexual dysfunction often can result in loss of self-esteem and a noted decrease in health-related quality of life (HRQoL). A recent study involving MS patients demonstrated that sexual dysfunction had a much larger detrimental impact on HRQoL parameters than did severity of physical disability, highlighting its key role in patients’ perceptions of well-being and the importance of incorporating it into clinical evaluations.^2^

Natalizumab (Tysabri®, Biogen, Cambridge, MA) is a disease-modifying therapy approved for the treatment of relapsing forms of MS.^4^ Beyond its effect on traditional MS clinical measures, it has been demonstrated that patients receiving natalizumab have improved HRQoL parameters, particularly fatigue and cognition.^5,6^ These positive treatment effects, in addition to our own anecdotal clinical experience utilizing natalizumab, have led us to hypothesize that there may be key improvements as a result of natalizumab administration with respect to sexual dysfunction, leading to an improvement in HRQoL. Here we report findings on patients’ perceived sexual dysfunction changes over 24 weeks after starting treatment with natalizumab.

## Study design and patients

This was a single-center, nonrandomized, open-label, single-arm, 24-week study conducted at our MS Center in Tampa, FL, USA. From 2011 to 2012, 45 patients were screened aged 18 to 65 years with relapsing forms of MS who were naïve to natalizumab and had a suboptimal response to, or tolerability issues with, other MS disease-modifying therapies. Inclusion criteria required patients to: (a) have stable disease as defined by not experiencing a MS relapse within previous 30 days before baseline, and (b) enroll in the Tysabri Outreach: Unified Commitment to Health (TOUCH®) Prescribing Program. Additional enrollment criteria included a required baseline level of 15 for the primary scale on the MS intimacy and sexuality questionnaire-19 (MSISQ-19) to demonstrate a clear level of sexual dysfunction due primarily to MS. In the original design we excluded patients with a moderate to high level of symptoms suggestive of depression as demonstrated by a score of ≥ 21 on the Beck depression inventory II (BDI-II). Due to the large number of screen failures due solely to this criterion, we removed it and analyzed the differences between baseline scores and post-treatment scores. Patients with major depressive disorder were excluded from the study. Other exclusion criteria included primary progressive or secondary progressive MS without relapses; treatment with medications known to aid in erectile dysfunction, such as phosphodiesterase-5 inhibitors, within 30 days of study initiation; and past medical history of diabetes mellitus. Patients who felt they would be unable to complete the study questionnaires were also excluded. The study was approved by the University of South Florida Institutional Review Board and conducted in accordance with the Declaration of Helsinki and International Conference on Harmonisation Guideline on Good Clinical Practice. All patients provided written informed consent.

## Methods

Enrolled patients received natalizumab (300 mg intravenously every 28 days) and completed evaluations of sexual dysfunction, QoL, depression, social and emotional well-being, mobility and fatigue at four visits at least 14 days apart, which included the screening/baseline visit (naïve to natalizumab therapy) and weeks 4, 12, and 24 after initiation of natalizumab therapy. The primary endpoint was change in the primary subscale of the MSISQ-19 score from baseline to week 24. The MSISQ-19 is a self-report patient-reported outcome (PRO) instrument for assessing the influence of MS symptoms on sexual activity and satisfaction. This patient questionnaire consists of 19 items divided into three subscales. There are five levels of responses (never, almost never, occasionally, almost always, and always) to each item based on its interference with sexual activity. The MSISQ-19 has demonstrated reliability and criterion validity in MS patients.^7,8^ The MSISQ-19 primary subscale demonstrates a direct physical effect that MS has on the physical component of sexual dysfunction. The secondary subscale of the MSISQ-19 cover questions about the patients’ other symptoms secondary to MS (such as spasticity, tremors, and concentration). The tertiary subscale then opens up the psychological aspects of sexual dysfunction. Secondary endpoints of the study included the change in the sexual function subscale of the multiple sclerosis quality of life-54 (MSQOL-54) questionnaire, the change in the overall quality of life subscale of the MSQOL-54, the change in score on the functional assessment of multiple sclerosis (FAMS) questionnaire, and the change in score on the BDI-II questionnaire over 24 weeks after treatment initiation. Two measures of HRQoL include the EuroQoL-5D questionnaire and the short form-36 patient questionnaire. Decreased health utility, and poor scores in other such domains on these questionnaires, have been shown to correlate with higher expanded disability status scale (EDSS) scores.^9–12^ EDSS is a means of grading disability in MS and tracking its progression over time. Although sexual dysfunction is not explicitly captured by the EDSS, a reduction in sustained disability progression as measured by the EDSS is seen with natalizumab as compared with placebo.^4^ The MSQOL-54 is a valid PRO self-report questionnaire that contains 52 items, distributed into 12 subscales, and two single items. The subscales are: physical function, role limitations physically and emotionally, pain, emotional well-being, energy, health perceptions, social function, cognitive function, health distress, overall QoL, and sexual function. The single-item measures are satisfaction with sexual function and change in health. The MSQOL-54 mental composite score includes five subscales measuring health distress, overall quality of life, emotional well-being, role limitations (emotional) and cognitive function. The MSQOL-54 sexual function subscale includes four gender specific questions that covered the physical aspects of sexual function. The FAMS questionnaire includes six sub-classes (mobility, symptoms, emotional well-being/depression, general contentment, thinking/fatigue, and family/social well-being) comprised of 59 items, 44 of which are used for scoring purposes. Each question is responded to with a ranking level from 0 (not at all) to 4 (very much). Higher scores on the FAMS indicate better QoL. The BDI-II is a widely used patient self-report inventory for depression. Patients choose one statement from each of 21 groups. Statements are ranked by severity level from 0 to 3; higher summative BDI-II scores indicate higher severity of symptoms suggestive of depression.

## Analysis of data

Baseline demographic and disease characteristics including age and gender were summarized using descriptive statistics. Baseline MSISQ-19, MSQOL-54, FAMS, and BDI-II scores were collected at a standard clinic visit at which the decision was made to initiate natalizumab for the treatment of relapsing MS. Due to the variation in insurance provider approval times, the data was collected anywhere from 14 days to more than 90 days prior to the first natalizumab infusion. Analyses were performed using the patient last follow-up visit in comparison to baseline scores; these were completed without imputation. Changes in sexual dysfunction measured over time per subject were analyzed using linear mixed modeling in SAS 9.4® software that accounted for non-independence of measures by treating subject as a random factor to define residuals for this model. Of the patients enrolled, 7 of the 15 completed all four time points of data collection (baseline, week 4, week 12 and week 24); the remaining 8 patients completed their last time point before week 24.

Analysis of covariance was used to assess associations between patient baseline characteristics and changes from baseline in QoL, depression symptoms and additional PRO scores at 24 weeks. To assess relationships between sexual dysfunction data and other characteristics of the patients (e.g., age, depression scores), Spearman correlation coefficients were determined for baseline data and for all post-baseline measurements. Analyses were performed using the intent-to-treat population; all patients with at least one completed follow-up assessment were included.

## Results

### Patient characteristics

**Figure 1.**
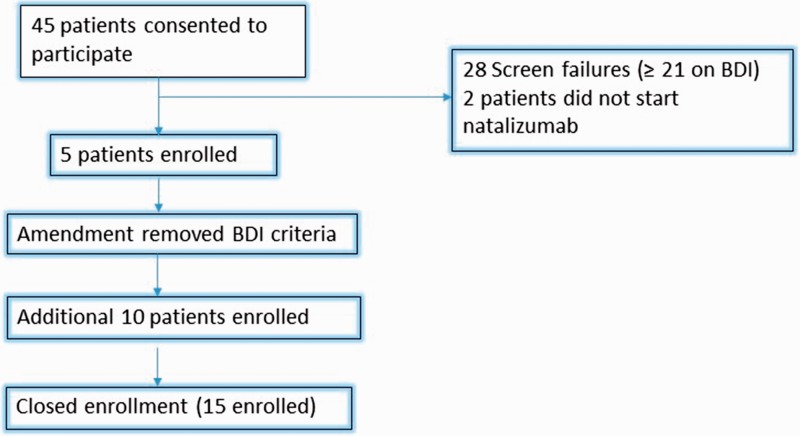
Participant recruitment and retention.

Of the 45 patients who were screened, a total of 15 patients were enrolled and were treated with natalizumab (Figure 1). The last 10 patients (of the total 15) were enrolled after the exclusion criteria was amended to allow inclusion of patients whose symptoms of depression scores were ≥21 on the BDI-II, as described previously. Patients who screened but did not continue were lost to follow-up (which included those who did not initiate therapy). Table 1 shows patient demographics and multiple sclerosis disease course information amongst study participants including past disease modifying therapy (DMT) history and usage. Glatiramer acetate had been the most widely used previous DMT prior to switching to natalizumab, followed by Interferon-beta-1a; 26% of participants were naïve to previous MS disease modifying therapy. The ratio of women to men was 3:1 as typically seen in the demographics of this disease. Median MS disease duration amongst all participants was 7 years.

**Table 1.** Subject demographics and disease characteristics of the study population (*n* = 15).

Characteristic	Value
Age, mean (years)	41.13
Disease duration, median (range)	7 (2–22)
Number of relapses in past year, mean ± SD	0.65 ± 0.573
Time (months) since most recent relapse, median (range)	3 (0–17)
Used prior MS treatment, %	73
Gender	
Male, n (%)	4 (26.7%)
Female, n (%)	11 (73.3%)
Prior MS treatment, %	
Interferon-beta-1a	20
Interferon-beta 1-b	7
Glatiramer acetate	40
Fingolimod	7
None	26

### Changes in sexual dysfunction and quality of life measures during treatment with natalizumab

The primary subscale of the MSISQ-19 questionnaire showed a statistically significant decrease in mean score of sexual dysfunction over time in patients on natalizumab therapy (–0.6976, *p* = 0.02), as shown in Table 2. The secondary and tertiary effects of MS on sexual function (such as spasticity, bowel/bladder symptoms, pain, and feelings of dependence or inadequacy), as measured by mean scores on the secondary and tertiary subscales of the MSISQ-19, did not meet statistical significance (0.019, *p* = 0.96; –0.048, *p* = 0.86). Other HRQoL scores analyzed for changes over time included the FAMS score (2.57, *p* = 0.01), the MSQOL-54 mental composite score (1.59, *p* = 0.08) and MSQOL-54 sexual function subscale (2.7, *p* = 0.09). Table 2 shows change in subjective, patient-reported quality of life metrics over the course of 6 months of natalizumab treatment including measurements of depression, functionality in daily life, sexual health, and overall quality of life. Overall the greatest positive change that achieved statistical significance over the course of the 6 months was seen on the FAMS, while the only negative change observed that achieved statistical significance during this time period was on the MSISQ, which are both positive clinical change indicators.

**Table 2.** Changes in quality of life scale scores from baseline to month 6.

Quality of life measure	Baseline mean	Change over 6 months	*p* Value
BDI, mean±SD	18.7 ± 11.9	–0.6 ± 0.48	0.22
FAMS, mean ± SD	86.7 ± 36.2	2.57 ± 0.99	0.01
MSISQ^a^, mean ± SD	17.5 ± 2.9	–0.697 ± 0.29	0.02
MSISQ^b^, mean ± SD	23.7 ± 6.0	0.019 ± 0.39	0.96
MSISQ^c^, mean ± SD	12.5 ± 4.7	–0.048 ± 0.28	0.87
MSQOL, mean ± SD	55.2 ± 18.3	0.73 ± 0.71	0.31
MSQOL^d^, mean ± SD	48.3 ± 21.5	1.59 ± 0.88	0.08
MSQOL^e^, mean ± SD	43.1 ± 24.5	0.29 ± 0.67	0.66
MSQOL^f^, mean ± SD	38.8 ± 26.1	16.1 ± 34.1	0.46

Follow-up for all patients did not occur at 6 months and may be calculated using last visit (1–6 month follow-up) in repeated measures model. BDI: Beck’s depression index; FAMS: functional assessment of multiple sclerosis; MSISQ: multiple sclerosis intimacy and sexuality questionnaire; MSQOL: multiple sclerosis quality of life.

^a^Primary sub-scale (items 12,16,17,18,19).

^b^Secondary sub-scale (items 1,2,3,4,5,6,8,10,11).

^c^Tertiary sub-scale (items 7,9,13,14,15).

^d^QOL as related to mental health.

^e^QOL as related to physical health.

^f^QOL as related to sexual function.

### Correlations between sexual dysfunction, age, and baseline depression symptom scores

Spearman correlation coefficients were not statistically significant for relationships between age and depression symptoms, age and primary sexual dysfunction, depression symptoms and primary sexual dysfunction, or gender and depression symptoms. These were also stratified by gender with no statistically significant differences noted. Due to one male patient being lost to follow up, only three were included in this analysis.

## Discussion

Sexual dysfunction in MS has unfortunately received relatively scant research evaluating its impact on HRQoL parameters, despite its high prevalence in MS patients.^5^ HRQoL metrics, in clinical studies and, to a lesser extent, in clinical practice, are being utilized more frequently to evaluate the impact of MS from the patient’s perspective, in addition to evaluation of treatment effects on HRQoL. It has been well demonstrated that natalizumab has many clinical benefits for patients with relapsing MS, including reduction in clinical relapses, slowing of the accumulation of disability, decrease in MRI lesion burden, improvement in fatigue, and improvement or stability of cognitive performance.^4,6,13^ To our knowledge, this was the first study to address sexual dysfunction changes in patients that are initiating natalizumab therapy. In our study we found that patients with known sexual dysfunction experienced a decrease in these symptoms while on natalizumab therapy, as demonstrated by a reduction on the primary subscale of the MSISQ-19. For example, the nearly 1-point improvement can translate from a sexual dysfunction symptom occurrence of “occasionally” reduced to “almost never” or another example from “almost always” to “occasionally” which has a very real potential to positively impact QoL. However, the small sample size was a limiting factor on the interpretation of our results. Therefore, an increase in sample size could have allowed for a more clinically meaningful improvement to be realized. They also experienced improved QoL overall while on natalizumab, as demonstrated by increased scores on the FAMS, a validated PRO tool for assessing HRQoL in MS.

Surprisingly, the secondary and tertiary subscale of the MSISQ-19 did not show significant change with natalizumab treatment. Although sexual dysfunction clearly can have a significant impact on HRQoL, the underlying etiology remains unclear. A prior study demonstrated an association between sexual dysfunction and sphincteric dysfunction in patients with MS, indicating that the same segments of the autonomic innervation controlling sphincteric function may be implicated in sexual dysfunction in these patients.^14^ Additionally, MS-related sexual dysfunction could be due to psychological factors such as depression and anxiety, fatigue, muscle weakness, muscle spasms, dysesthesias and pain, and side effects of commonly prescribed symptomatic medications including anticonvulsants, antidepressants, and bladder medications.^15^ There is an established high prevalence of sexual dysfunction in those with severe depression.^16^ Given this potential confounding variable, we excluded patients with a moderate to high level of depression symptoms as demonstrated by a score of ≥ 21 on the BDI-II. This criterion led to a high number of screen failures for patients attempting to enroll in the study therefore a protocol amendment was implemented to remove this exclusion criterion. We analyzed the differences between baseline scores and post-treatment scores. The results were not statistically significant for relationships between depression symptoms scores and primary sexual dysfunction.

Our study had several limitations that prevent any definitive conclusions about the effects of natalizumab on sexual dysfunction. First, we had a relatively small sample size. Second, due to variability in scheduling of the natalizumab infusions, complete data capture for all study patients was difficult. Approximately 50% of patients completed all protocol specified follow-up and nearly 75% of patients had complete data through week 12. Third, the lack of a placebo group or active comparator arm in the study design also contributed to the interpretation of the results. These limitations would be a potential explanation as to why the primary subscale of MSISQ-19 did show a statistically significant reduction with initiation of natalizumab therapy while the secondary and tertiary subscale of the MSISQ-19 did not.

## Conclusion

Our study results strengthen the growing evidence that PROs have an important role in measuring the effects of various treatments for MS, including natalizumab. Standard outcome measures that have routinely been utilized in making treatment decisions for MS patients include neurologic disability, relapse history, and MRI lesion burden. HRQoL metrics, such as PROs, record the impact of the disease from the patient’s perspective and should be considered by healthcare providers in conjunction with these standard outcome measures. Given its high prevalence in MS and its significant impact on patient-reported HRQoL, sexual dysfunction could benefit from further research; specifically, more studies are needed to assess the relative effects of current MS treatments on sexual dysfunction and QoL.

## Disclosures

Derrick Robertson has been a consultant for Biogen, EMD Serono, Genentech, Genzyme, Novartis, Pfizer, and Teva; has received honoraria or speaker’s fees from Acorda, Biogen, EMD Serono, Genentech, Genzyme, Mallinckrodt, Novartis, Pfizer, and Teva; and has received research grant support from Actelion, Biogen, EMD Serono, Genentech, Genzyme, Mallinckrodt, MedImmune, Novartis, and Sun Pharma. Lise Casady has received honoraria or speaker’s fees from Genzyme, Biogen and Genentech. All other authors report no conflicts of interest.
